# Climate, intrinsic water-use efficiency and tree growth over the past 150 years in humid subtropical China

**DOI:** 10.1371/journal.pone.0172045

**Published:** 2017-02-09

**Authors:** Dawen Li, Keyan Fang, Yingjun Li, Deliang Chen, Xiaohong Liu, Zhipeng Dong, Feifei Zhou, Guoyang Guo, Feng Shi, Chenxi Xu, Yanping Li

**Affiliations:** 1 Institute of Geography, Key Laboratory of Humid Subtropical Eco-Geographical Process (Ministry of Education), College of Geographical Sciences, Fujian Normal University, Fuzhou, China; 2 Regional Climate Group, Department of Earth Sciences, University of Gothenburg, Gothenburg, Sweden; 3 Northwest Institute of Eco-Environment and Resources, Chinese Academy of Sciences, Lanzhou, China; 4 Key Laboratory of Cenozoic Geology and Environment, Institute of Geology and Geophysics, Chinese Academy of Sciences, Beijing, China; Pacific Northwest National Laboratory, UNITED STATES

## Abstract

Influence of long-term changes in climate and CO_2_ concentration on intrinsic water-use efficiency (iWUE), defined as the ratio between net photosynthesis (*A*) and leaf conductance (*g*), and tree growth remain not fully revealed in humid subtropical China, which is distinct from other arid subtropical areas with dense coverage of broadleaf forests. This study presented the first tree-ring stable carbon isotope (δ^13^C) and iWUE series of *Pinus massoniana* from 1865 to 2013 in Fujian province, humid subtropical China, and the first tree-ring width standard chronology during the period of 1836–2013 for the Niumulin Nature Reserve (NML). Tree-ring width growth was limited by precipitation in July-August (r = 0.40, p < 0.01). The tree-ring carbon isotope discrimination (Δ^13^C) was mainly controlled by the sunshine hours (r = -0.66, p < 0.001) and relative humidity (r = 0.58, p < 0.001) in September-October, a season with rapid latewood formation in this area. The iWUE increased by 42.6% and the atmospheric CO_2_ concentration (*c*_*a*_) explained 92.6% of the iWUE variance over the last 150 years. The steady increase in iWUE suggests an active response with a proportional increase in intercellular CO_2_ concentration (*c*_i_) in response to increase in *c*_a_. The contribution of iWUE to tree growth in the study region is not conspicuous, which points to influences of other factors such as climate.

## Introduction

Global warming and increase in CO_2_ concentration have profound influences on forests growth by directly modulating their physiological processes, e.g. photosynthesis, and indirectly by changing the forests structures. These changes can in turn feedback climate change via modulating carbon cycles since forests are important carbon sink [1–3]. Interactions among temperature, CO_2_ concentration and forests growth vary from region to region, which is largely because of the important roles of regional factors. This is largely because that global increase in temperature and CO_2_ concentrations can lead to regionally different changes in hydrological patterns, which cause varying degrees of water stress [4, 5]. Meanwhile, it can also lead to regionally different changes in intrinsic water-use efficiency (iWUE). For example, the increase in CO_2_ concentration in the arid region is expected to facilitate tree growth because that the increase in iWUE in response to an increase in CO_2_ concentration can alleviate the drought stress for tree growth [6]. However, the climate-iWUE-tree growth linkages can be complex in humid and hot regions due to the relatively “satisfied” environments for tree growth. For example, in Brazil, tree growths in some tropical regions are enhanced in responses to increase in temperature and CO_2_ concentration, while tree growths in other tropical regions such as Bolivia and Thailand are not insensitive to changes temperature and CO_2_ concentration [5, 7–8].

The study region is located in southeastern China, which holds the world’s largest subtropical evergreen broadleaf forests due to its humid subtropical climate, which is distinct from other arid regions of the subtropics [9]. Although annual precipitation is abundant for this area, previous studies revealed summer drought stress for tree growth in this area due to the relatively hot and dry climate in that season [10–12]. However, little is not known about the variations of iWUE and its potential influence on tree growth in this region. This study attempts to improve our understandings of the linkages among climate-iWUE-tree growth in the humid subtropical China.

Monitoring studies, such as laboratory, free-air CO_2_ enrichment (FACE) experiments and process-based simulations, frequently suggested promotion of tree growths in response to an increase in CO_2_ concentration [13, 14]. However, other study revealed that trees may adapt to the high CO_2_ concentration environments over a long period, thus becoming insensitive to CO_2_ increase [6]. Therefore, it is crucial to not only understand the short-term responses of forests growth to environmental change but also to study their long-term adaptions to environmental stress. Tree-ring width is an invaluable indicator to infer radial tree growth in a long period of decades to centuries, which has been widely used to study the responses of forests growth to climate change [15, 16]. Due to the high sensitivity of tree-ring data to climate change, tree-ring proxy records of precipitation and temperature are one of our best indicators for how past climate change has impacted ancient human societies. The stable carbon isotope ratio (δ^13^C) represents the ratio of carbon assimilation to water costs through transpiration, which is can be used to infer the iWUE changes [17–19]. Thus tree-ring δ^13^C has been widely used to study iWUE changes and their relationships with tree growth and environmental factors [14, 18, 20, 21]. In addition, relative to the tree-ring width data, tree-ring δ^13^C data have higher signal to noise ratio and are less influenced by age related growth trends [22]. Thus tree-ring δ^13^C data have been widely used to reconstruct long-term changes of various environmental factors including sunshine, temperature and humidity [23–25].

Currently, most of the tree-ring based climate studies in China concentrated in the arid and cold northwestern parts [24, 26–35]. Although there were some tree-ring based reconstructions conducted in the humid subtropical China [36–38], the tree-ring network for the humid subtropical China in the southeast is still much sparser than the northwest [39], particularly for the tree-ring based isotopic studies. For example, for our study region in Fujian province, there is currently only one tree-ring oxygen isotopic series published in its west [40]. This study presents the first tree-ring δ^13^C series of *Pinus massoniana* trees for Fujian province and the first tree-ring width chronology for Niumulin Nature Reserve (NML). The newly developed tree-ring and iWUE data will be compared with changes in climate and atmospheric CO_2_ concentration to explore their relationships.

## Materials and methods

### Study region

**Fig 1:**
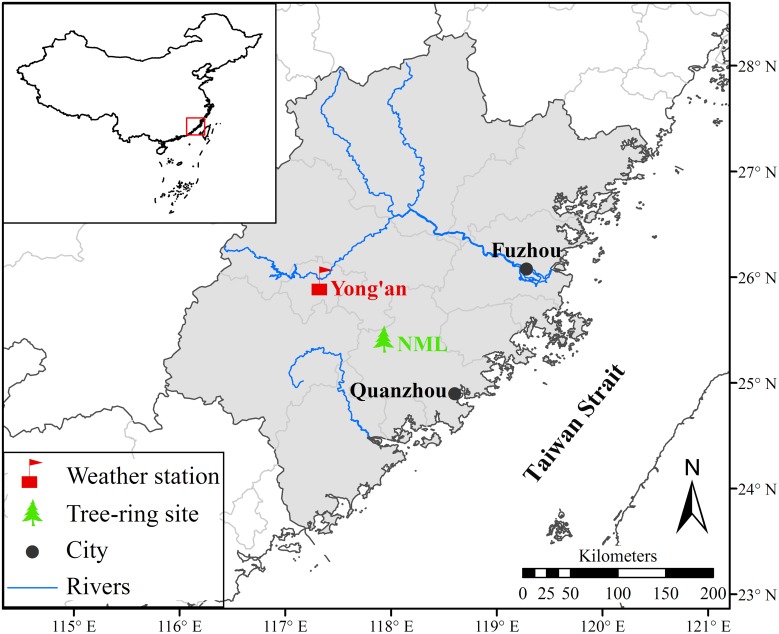
Locations of the tree-ring sampling site, the meteorological station and the close city in eastern Fujian province. The maps were derived from Geographic Information Resources Service (http://www.webmap.cn/mapDataAction.do?method=forw&resType=5) and produced using ArcMap 10.0 software.

**Fig 2:**
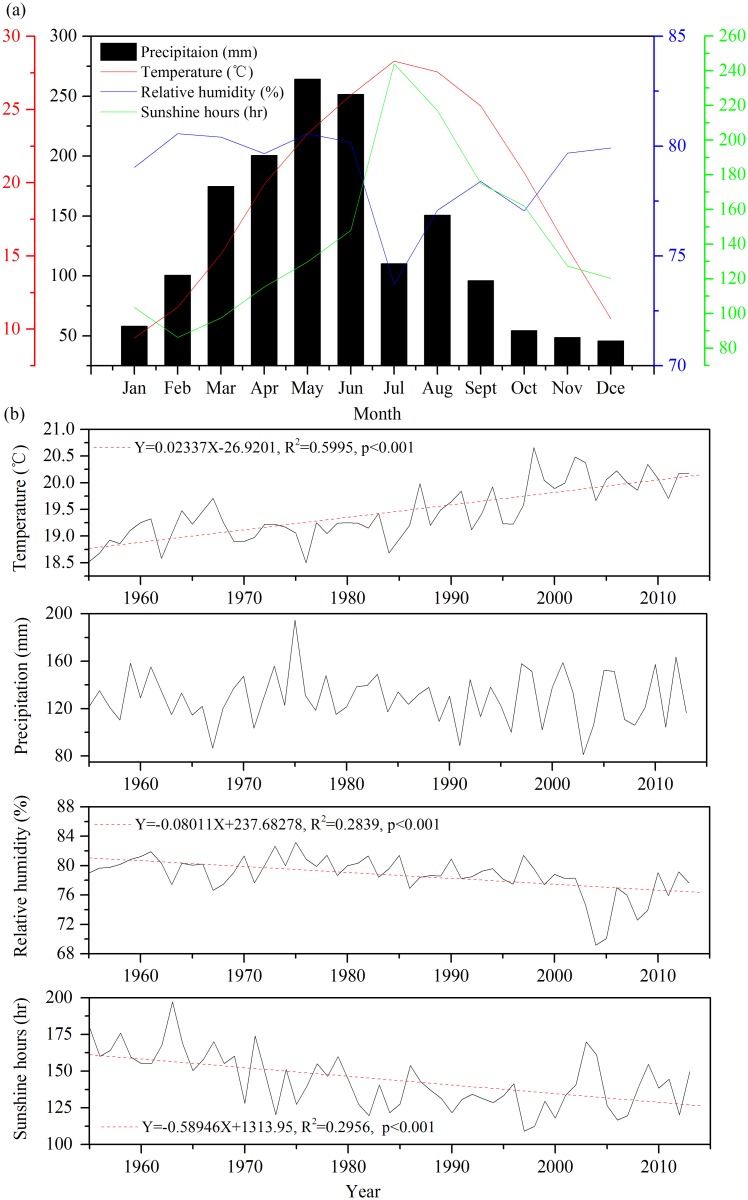
(a) Monthly climatic diagram at the Yong’an meteorological station (25°58’N, 117°21’E, 206 m a.s.l.) near the sampling site. Time series of the (b) mean temperature, (c) precipitation, (d) relative humidity and (e) sunshine hours for the period of 1955–2013. The red dashed line indicates the significance level (p < 0.001) for linear regression.

Tree-rings were sampled in NML (25°25’N, 117°56’E) Nature Reserve of Quanzhou area, southeastern Fujian province of China, which is western to the Taiwan Strait (Fig 1). The NML is a branch mountain of Daiyun Mountain range, the major mountain range of central Fujian province [11]. The study region is mainly covered by relatively acidic red and yellow soil with a thick humus layer on its top. The area is characterized by a hot and humid monsoon climate with the mean monthly temperature ranging from 9.4°C in January to 28.3°C in July. The mean annual temperature is 19.4°C over the period of 1955–2013 according to instrumental data from the nearest meteorological station of Yong’an (25°58’N, 117°21’E, 206 m a.s.l.). The annual total precipitation is 1556 mm, and 87% of the precipitation concentrates in the growing season from March to November (Fig 2a). Different from other monsoonal areas of China, precipitation in the hottest July does not correspond to the highest precipitation due to the control of the western Pacific High in this season [11, 12]. The annual mean temperature shows a significant increasing trend during the period of 1955–2013 (Fig 2b), while relative humidity and sunshine hours significantly decrease during study period (Fig 2d and 2e). The annual precipitation has no obvious trend (Fig 2c). The study area is dominated by evergreen broadleaf forest, although some sites, including our sampling site, have relatively pure *Pinus Massoniana* forests mixed with bamboo. The tree-ring cores were taken from the old-growth *Pinus Massoniana* trees with diameter of ~2–3 meters at the breast height.

### Tree-ring width data

According to standard dendrochronological methods [15], we sampled 72 cores at 1.3m breast height from 40 dominant living *Pinus massoniana* trees from NML Nature Reserve using 5-mm diameter increment borer. The sampling work was conducted under the permission of the administrators of the natural reserve. The cores were air-dried and polished by a series of sand-paper until the cellular structure is clearly visible [41]. Calendar years were assigned to each ring by checking the matches of the tree-ring patterns following cross-dating procedure, which were further checked by COFECHA program for quality control [42]. The cross-dated series were measured to a 0.001mm precision using a LINTAB 6.0 measuring station. Age related growth trends of the raw measurements were removed by fitting a smoothed cubic spline curves with two-thirds of the mean lengths of each series. The detrended tree-ring indices were then averaged to produce the tree-ring chronology following the biweight robust mean methods using ARSTAN program [43]. We selected standard chronology (STD) because we are interested in both low- and high-frequency variations and the STD contains more low-frequency signal than residual chronology. To account for the decreased sample number in the early period of the chronology, we used the statistic of the Expressed Population Signal (EPS) over 0.85 to determine the reliable portion of STD [44].

### Tree-ring δ^13^C, Δ^13^C and iWUE

We selected twelve cores from twelve trees without absent rings and obvious growth disturbance for stable isotopes analysis, covering the period of 1844–2013. The whole wood of each ring was pooled to extract their α-cellulose following standard methodology [21, 45]. The pooled samples of the same year were dried for 72 hours at 75°C in the oven and then milled in a grind bowl (Pulv-erisette 23, Fritch, Germany) until the samples can pass through the 60-mesh sieve. We then treat the dried out samples to [46, 47]: (1) remove lipid by organic solvents (“Acetone” and “Toluene/Ethanol 2:1”); (2) remove lignin by sodium hypochlorite and acetic acid solutions; (3) remove hemicellulose and decomposed lignin by sodium hydroxide solutions. To obtain highly homogeneous α-cellulose, an ultrasound machine (JY92-2D, Ningbo Scientz Biotechnology, Ningbo, China) was used to break the cellulose fibers [48].

We packed 0.10–0.12 mg of α-cellulose in tin capsules for stable isotope measurement using the Flash Elemental Analyzer (Flash 2000) coupled with a Thermo Scientific MAT 253 (Thermo Electron Corporation, Bremen, Germany). Each sample was repeatedly measured for two to four times. The Charcoal Black (standard sample, δ^13^C = -22.43‰) was used to calibrate the values of δ^13^C gained from tree-ring α-cellulose. For convenience, the rate of stable carbon isotope (^13^C/^12^C) was defined in delta (δ) according to the Vienna Pee Dee Belemnite (VPDB) standard [49], in parts per thousand (‰):

$$\delta^{\text{13}}\text{C}=\left[\left({\text{R}}_{\text{sample}}/{\text{R}}_{\text{standard}}\right)-1\right]\times 1000$$ (1)

where R_sample_ and R_standard_ represent the ^13^C/^12^C ratios of tree-ring α-cellulose sample and VPDB standard, respectively. Our isotopic measurements were stable with a low standard deviation of 0.07‰.

The stable carbon isotopes discrimination (Δ^13^C) in C_3_ plants indicates the isotope changes between atmosphere and plants (i.e. leaf gas-exchange), which can be expressed as the following equation [1]:

$$\Delta^{13}C=\left(\delta^{13}C_{a}-\delta^{13}C_{p}\right)/\left(1+\delta^{13}C_{p}/1000\right)=a+\left(b-a\right)\left(c_{i}/c_{a}\right)$$ (2)

where △^13^C is the stable carbon isotope discrimination; δ^13^C_a_ and δ^13^C_p_ are the stable carbon isotopic ratios (^13^C/^12^C) of ambient air and plant cellulose; a (≈ 4.4‰) represents the isotope discrimination of atmospheric CO_2_ entering in the intercellular space; b (≈ 27‰) represents the isotope discrimination value due to the carboxylation; *c*_*i*_ and *c*_*a*_ are the intercellular and ambient CO_2_ concentrations, respectively. We assumed that the values of *c*_*a*_ and δ^13^C_a_ at the sampling site are equal to the atmospheric CO_2_ concentration and its carbon isotope, respectively, as previous studies revealed.

The intrinsic water-use efficiency (iWUE) is defined as the ratio of the fluxes of net photosynthesis and conductance for water vapor, which indicates the cost of assimilation per unit of water, expressed in the units of umol mol^-1^ [17]:

$$iWUE={A/g}_{s}=\left(c_{a}-c_{i}\right)/1.6=c_{a}\left(1-c_{i}/c_{a}\right)/1.6$$ (3)

where A is the rate of CO_2_ assimilation and g_s_ is the rate of leaf stomatal conductance.

### Climate data and analytical methods

We employed the instrumental data of monthly mean temperature, sunshine hours, monthly total precipitation and monthly mean relative humidity from the Yong’an meteorological station. The Pearson correlations among climate, tree-ring width, tree-ring Δ^13^C and iWUE were calculated using both the raw and the first-order differenced data. The first-order differenced data were calculated as the residual between the tree-ring data of successive years. The correlations based on the first-order differenced data are used to highlight the interannual variability of tree rings. All the correlation analyses between tree-ring data and climate variables were calculated from the start of previous growing season (i.e. previous February) to the end of the current year (current December). Apart from the calculations of the Pearson correlations, we calculated the response function analysis using the program DendroClim2002, which employs the bootstrap procedure to evaluate the significance level [50].

## Results

### Tree-ring width chronology and climate-growth relationship

**Fig 3:**
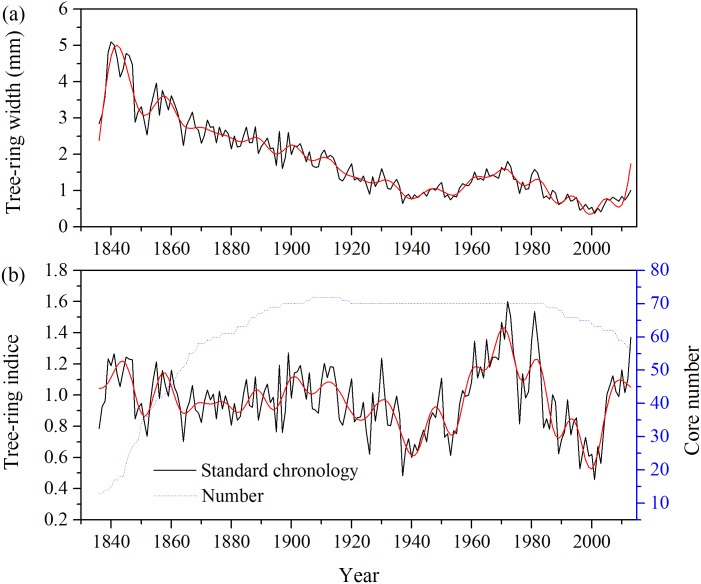
Time series of the (a) raw tree-ring width chronology and (b) tree-ring standard chronology (STD) and the sample size from *Pinus massoniana* of the Niumulin Nature Reserve (NML) for the period of 1836–2013. The reliable period of the chronology with over 13 cores is determined with the static of Expression Population Signal (EPS) greater than 0.85. The red lines represent the result of FFT filtering using a 10-year window to emphasize the low-frequency variations.

It is expectable to observe a lapsing trend for the tree-ring width from NML site over the period of 1836–2013 due to the increase in tree diameter. However, increasing trends are also observed in the 1940s-1970s period and the recent 10 years (Fig 3a). After removing the age-related growth trend (Fig 3b), the tree-ring width standard chronology (STD) were relatively stable before 1910s with weak interdecadal variability. The interdecadal variability of the STD enhanced after the 1910s with a peak in the 1970s and a growth decline from 1980s to 1990s.

**Fig 4:**
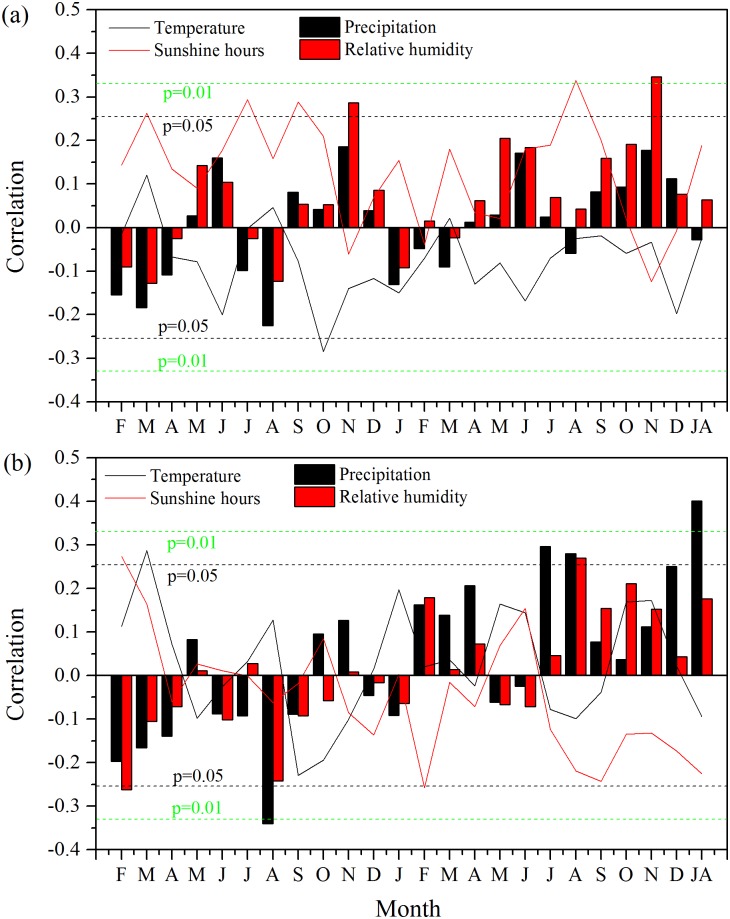
Correlations between climate variables and (a) the tree-ring standard chronology (STD), and (b) the first-order differenced STD from the start of previous growing season (previous February) to the end of current growing season (current December) for the period of 1955–2013. JA represents the mean between July and August. The black (green) horizontal dash lines represent the 95% (99%) confidence level.

As shown in Fig 4a, the STD was significantly negatively correlated with temperature in previous October, but there was no significant correlation with precipitation. Significant positive correlations between STD and relative humidity were seen in previous November and current November. The STD was positively and significantly correlated with sunshine hours in previous February, July, September, as well as current August. The first-order differenced STD was positively and significantly correlated with temperature in previous March (Fig 4b). The first-order differenced STD was significantly negatively correlated with precipitation in previous August and significantly positively correlated with precipitation in current July and August. Significant negative correlations with relative humidity were found for first-order differenced data in previous February and current August. Positive correlations with sunshine hours are observed for the first-order differenced data in previous February and negatively correlated with sunshine hours in current February were also seen in the correlations. The highest response for the first-order differenced data was found with monthly total precipitation in current July-August (r = 0.40, p < 0.01).

### Tree-ring δ^13^C and response of Δ^13^C to climate change

**Fig 5:**
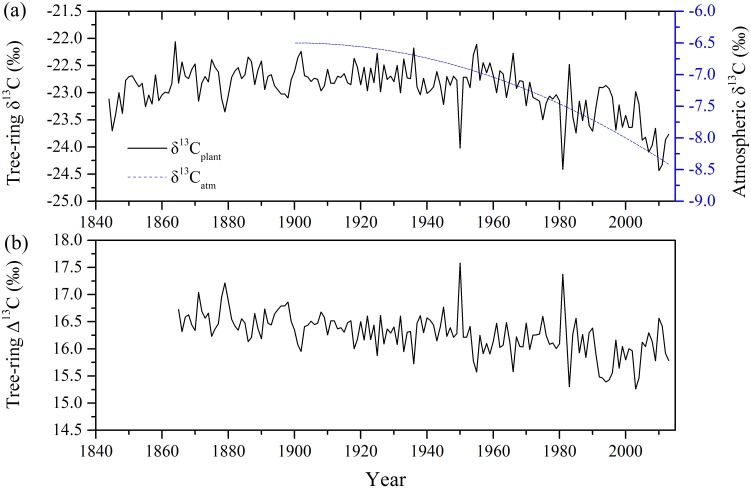
(a) Tree-ring carbon isotope ratio (δ^13^C_p_) for the *Pinus massoniana* in Niumulin Nature Reserve (NML) during 1844–2013 and atmospheric carbon isotope ratio (δ^13^C_a_) from 1900 to 2013, and (b) tree-ring carbon isotope discrimination (△^13^C) during 1865–2013. The values of δ^13^C_a_ were derived from Francey et al [52].

The tree-ring δ^13^C (δ^13^C_p_) chronology spans 170 years from 1844 to 2013. An increasing trend in δ^13^C_p_ was observed over the first 20 years of the chronology from 1844 to 1864, which may be caused by the “juvenile effect” (Fig 5a). Juvenile trees tend to assimilate more CO_2_ from soil respiration and can cause an increase in δ^13^C_p_ [2]. To avoid the juvenile effect, we discarded the data over first 20 years and only selected the δ^13^C_p_ data from the time interval from 1865 to 2013 for further analyses. Human activities such as fossil fuel burning and deforestation, referred to as “Suess effect”, have resulted in significantly decreasing trend in δ^13^C of atmosphere, leading to a decreasing trend in δ^13^C_p_ since the industrial era [2, 51, 52]. The δ^13^C_p_ series showed an obvious decreasing trend (a slop of linear regression: -0.006) over the study period. This indicates that the decreasing trend is likely due to the trend caused by the decrease in atmospheric δ^13^C since the beginning of the industrial era. However, the conspicuous decreasing trend is not observed in tree-ring Δ^13^C series (Fig 5).

**Fig 6:**
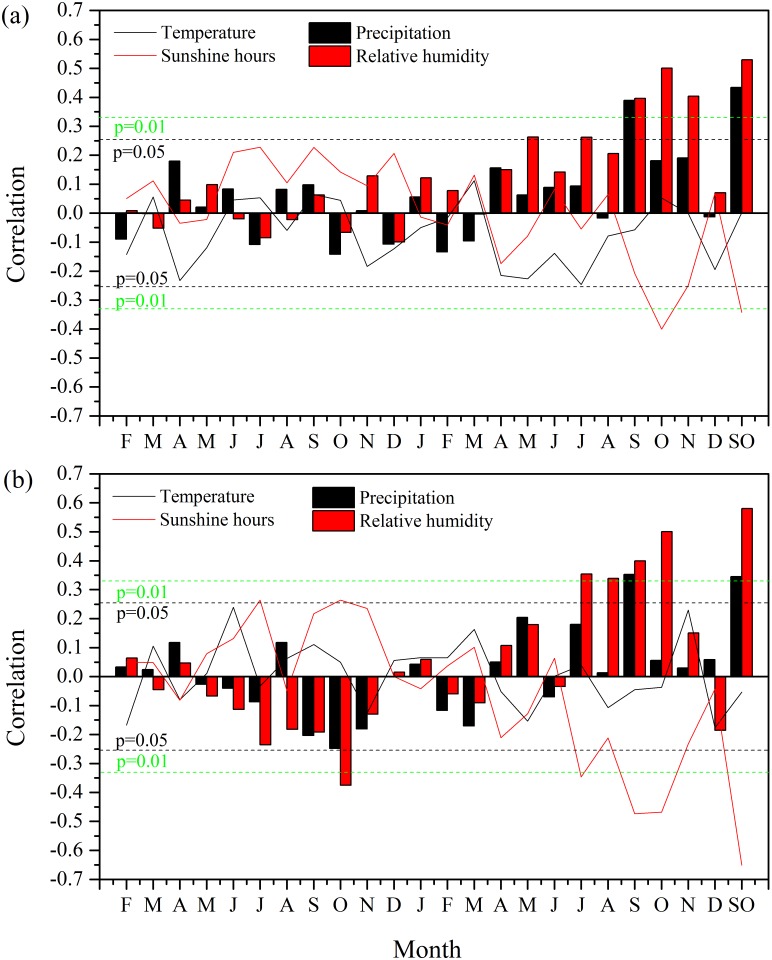
Correlations between climate variables and (a) the series of tree-ring carbon isotope discrimination (△^13^C), and (b) the first-order differenced △^13^C from the start of previous growing season (previous February) to the end of current growing season (current December) for the period of 1955–2013. SO represents the combination of data from September to October. The black (green) horizontal dash lines represent the 95% (99%) confidence level.

The tree-ring △^13^C has significantly positive correlation with precipitation in current September (Fig 6a). Peak correlation between the △^13^C and relative humidity was found in October (r = 0.50, p < 0.001). In addition, the △^13^C was also significantly and negatively correlated with sunshine hours in October and November. Correlations among the △^13^C and climate factors based on first-order differenced data were similar to those derived from the raw data (Fig 6b). The correlations with relative humidity and sunshine hours in September-October were higher than those derived from the raw data (Fig 6).

### Trends in *c*_*a*_, *c*_*i*_, *c*_*i*_/*c*_*a*_ and iWUE

**Fig 7:**
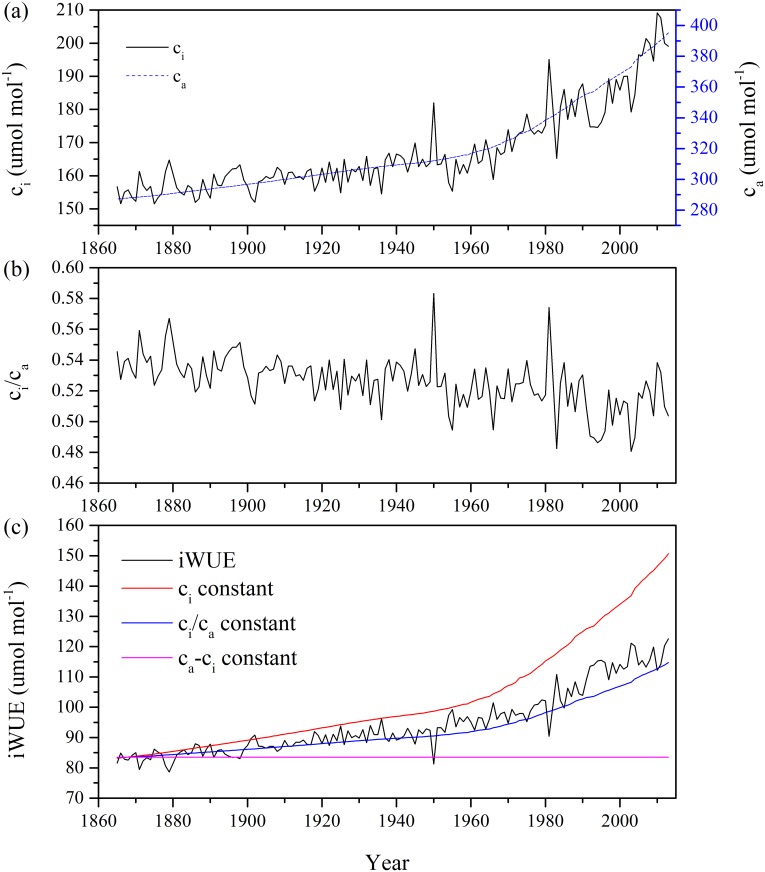
Variations in (a) leaf intercellular CO_2_ concentration (*c*_*i*_) and ambient CO_2_ concentration (*c*_*a*_), (b) ^13^C discrimination ratios (*c*_*i*_/*c*_*a*_) and (c) intrinsic water-use efficiency (iWUE) calculated from three scenarios as a baseline for interpreting the gas exchange in response to increasing *c*_*a*_ over the period of 1865–2013. The atmospheric CO_2_ were derived from McCarroll and Loader [2].

**Fig 8:**
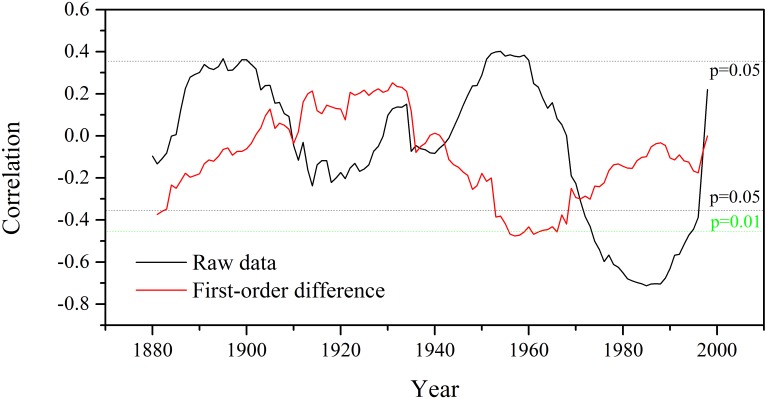
Running correlations based on a 31-year window of the tree-ring standard chronology and intrinsic water-use efficiency (iWUE). The study viewed intermediate point as a starting year. The black (green) horizontal dash lines represent the 95% (99%) confidence level.

Overall, the *c*_a_ rose from 287.2 ppm to 395.3 ppm from 1865 to 2013. The *c*_i_ calculated from tree-ring δ^13^C ranged from 151.5 ppm in 1875 to 209.2 ppm in 2010, showing a slightly increasing trend than *c*_a_ (Fig 7a). Accordingly, the ratio between *c*_i_ and *c*_a_ showed a significant decreasing trend (Y = −0.0002X + 0.54, R^2^ = 0.30, p < 0.001, X is the order of year) during the study period (Fig 7b). The iWUE increased by about 42.6% during the study period (Fig 7c). The *c*_a_ explained 86.9% and 92.6% of the increase in *c*_i_ and iWUE during the period of 1865–2013 based on the regression analysis, respectively. It suggested that the increased atmospheric CO_2_ concentration was the major contributor to the *c*_i_ and iWUE increases. There was a gradual increase in *c*_i_, *c*_a_ and iWUE before ~1960s, while the increasing trend became more conspicuous afterwards. Since both *c*_i_ and *c*_i_/*c*_a_ had slightly increasing trends, a relative constant iWUE since 2003 was observed (Fig 7). The iWUE had significant (p < 0.05) and positive correlations with tree growth in ~1890s and ~1950s but negative correlations in ~1970s-90s (Fig 8). Significant (p < 0.05) and negative correlations are only seen in ~1880 and ~1950s-60s (Fig 8) for the first-order differenced data.

## Discussion

### Summer drought stress and tree-growth

The correlation analysis for the first-order difference data revealed a drought stress in summer in July and August on tree growth in our site, which is not conspicuous for the correlations using raw data (Fig 4). We considered that the correlations using the first-order difference data should be more robust as they are less affected by the trends and often have a higher effective degree of freedom due to their high-frequency variability. In addition, correlations with the first-order difference data often have more significant correlations with precipitation but less so with temperature, compared with the raw data. This is likely because the precipitation tends to have more conspicuous high-frequency variability while the temperature tends to have relatively strong low-frequency variability [53]. The summer drought stressed growth patterns have also been revealed in nearby regions of eastern Fujian province. For example, Li et al. [12] revealed that the growths of tree-rings of *Pinus massoniana* were mainly controlled by the July-September precipitation in Fuzhou area in the eastern of Fujian province. Chen et al. [11] also demonstrated that the July-August precipitation is the major limiting factor for tree-ring growth of *Pinus taiwanensis* in Daiyun Mountain of Quanzhou area, southeastern of Fujian province. The presence of the summer drought stress is due to the fact that the peak temperature in July and August was accompanied by relatively low precipitation in this period (Fig 2a). The hot climate in summer may lead to stomatal closure and an increase in evaporation from soil, causing a decrease in water supply for tree growth. In such conditions, a relatively low precipitation can be a limiting factor for tree growth.

This study did not reveal significant positive response to winter temperature as other studies in southeastern China [38]. This may be because our sampling site is not located at the high altitude sites as Chen et al. [11], where the winter temperature are too low to limit tree growth. The summer drought stressed growth patterns in humid subtropical China are different from the drought stressed pattern in arid western China [16], where a significant and negative response to summer temperature was often the case. This is because that warming induced evapotranspiration in the arid region can be more stressful for vegetation growth than the humid region with relatively abundant precipitation.

### Modulations of insolation and relative humidity on tree-ring Δ^13^C

Both the raw and first-order differenced tree-ring Δ^13^C have negative correlations with insulation and positive correlations with relative humidity in September-October (Fig 6). This is likely due to a tradeoff between photosynthetic rate and stomatal conductance in tree-ring Δ^13^C [1]. McCarroll and Loader [2] found that the direct control of carbon isotope discrimination was photon flux, which modulates the photosynthetic rate. The *c*_i_ decreased when the amount of photosynthesis strengthened, leading to the decrease of trees stable carbon discrimination. This accounts for the negative correlations between Δ^13^C and sunshine hours (Eq 2) [1]. The tree-ring Δ^13^C has also been found to be sensitive to insulation in northwestern Norway and the Slovenian Alps [23, 54]. Temperature is another factor modulating photosynthesis and thus sensitive to carbon isotope discrimination revealed in some other studies [2, 24]. Relative to temperature, insulation and photon flux have more direct influences on photosynthesis and tree-ring Δ^13^C [2, 23]. As for this hot and humid area, the temperature may be sufficient for photosynthesis while the insulation can be insufficient due to prevailing of cloudy days. For example, a study in Taiwan, neighboring to our study region, also found that sunshine may be the key limiting factor for tree growth [55].

On the other hand, precipitation and relative humidity indirectly modulates the tree-ring carbon isotope discrimination by decreasing stomatal conductance in response to moisture stress [18, 56]. The low relative humidity will induce the decrease of stomatal conductance for trees to keep alive. Then *c*_*i*_ decreased, to induce the decrease of tree-ring Δ^13^C. This accounts for the positive responses of tree-ring Δ^13^C to relative humidity at our site (Eq 2) [1]. Another study in western Fujian found limitation of precipitation on tree-ring oxygen stable isotope, which may reflect the stomatal conductance [40]. We also observed the influences of precipitation and relative humidity on the growths of tree-ring widths in this area.

Both of the responses of tree-ring widths and Δ^13^C are sensitive to relative humidity/precipitation. However, the response of tree-ring Δ^13^C has a more conspicuous delayed response to moisture in autumn instead of in summer for tree-ring width. Different responses of the two parameters may be because of the different processes determining the tree-ring width and carbon isotope discrimination, which are mainly modulated by photosynthesis and respiration and by photosynthesis and stomatal conductance [1, 2, 18]. In autumn, the sunshine hours are only secondary to the summer (July and August) (Fig 2a). The season has high photosynthesis rate due to the high sunshine hours and moderately high temperature, while the stomatal conductance may not be high due to the relatively low precipitation. This may result in a high ratio between photosynthesis rate and stomatal conductance, which can cause high tree-ring Δ^13^C. High tree-ring Δ^13^C in autumn may contribute to its high sensitivity to climate in this season. This period was also found to be associated with a rapid growth of latewood of *Pinus massoniana* in this area [57]. Our hypothesis needs to be tested by interannual monitoring studies on tree growth and isotopic discrimination.

### Responses of iWUE to CO_2_ increase and its relationships with tree-growth

Our study suggested a dominant control of *c*_a_ on iWUE. Three scenarios were proposed by Saurer et al. [18] to account for the responses of iWUE to changes of *c*_a_ as follows (Eq 3): (1) *c*_i_ remains constant with an intense decrease in *c*_i_/*c*_a_ and increase in iWUE; (2) *c*_i_ and *c*_a_ increase in the same proportion, i.e. a constant *c*_i_/*c*_a_, and iWUE increase, representing an active response of an increase in iWUE to *c*_a_ increase; (3) *c*_i_ and *c*_a_ increase in the same amount, i.e. a constant *c*_a_-*c*_i_, representing a passive response with no changes in iWUE. The second scenario was significantly related to iWUE in the NML site, which indicated that the trees actively responded to *c*_a_ before 1970s (Fig 7c). Similarly active responses were found from the high latitudes, tropical and arid areas [8, 20, 21]. The increase in iWUE in response to *c*_a_ may have been caused by the *c*_a_ induced increase in CO_2_ assimilation and/or stomatal closure which were the major control factors in influencing the variation of iWUE, according to its definition [1, 2]. This has been evidenced by some control experiments [13, 14]. iWUE became close to the *c*_i_ scenario from 1980s to 2003, representing strongest response to *c*_a_ increase (Fig 7c). Similarly, the physiological response of trees switched to a near *c*_a_-*c*_i_ constant scenario with constant iWUE after 2003, representing a passive response to *c*_a_, which has also been revealed in previous studies [14, 19, 58] (Fig 7c). The *c*_a_ accounts for the centennial trends of the iWUE, while the interannual and decadal variations of iWUE may have been caused by other factors such as climate. Previous studies suggested that trees may switch to passive response when water stress becomes significant [19, 22]. The relative constant iWUE since 2003 may have resulted from the relative low water stress as the relative humidity increased in recent years (Fig 2d). This can cause an increase of *c*_i_, and thus a constant *c*_a_-*c*_i_ and iWUE. It may also be related to other factors such as air pollution [14], which can increase the *c*_i_ via increased stomatal conductance in relatively clean region [59]. In addition, *c*_a_ may have reached a threshold in causing in iWUE since 2003 [60]. Over the whole period, the iWUE showed slightly increasing trend before ~1960s (slop of linear regression: 0.13) and rapidly increasing trend afterwards (slop of linear regression: 0.53), indicating that iWUE of the NML site was mainly influenced by rapidly increased *c*_a_ since 1960s [61].

The iWUE and tree-ring Δ^13^C are negatively related according to their definitions (Eqs 2 and 3), and iWUE has positive correlations with sunshine hours and negative correlations with relative humidity (S1b Fig). Although there are periods with positive correlations between iWUE and tree growth, significant (p < 0.05) correlations have not been found between them based on the first-order difference data (Fig 8). Thus, it is still not reliable to confirm the positive correlations between iWUE and tree growth during those periods since these positive correlations may be due to the similarly interdecadal trends caused by interdecadal climate change. Although there were studies revealing contribution of the increase in iWUE on tree growth [18, 20], numerous studies revealed no promotions of iWUE on tree growth in various regions. For example, Penuelas et al. [62] showed that increased iWUE for the major forest biome types from 47 sample sites all over the word did not lead to enhanced tree growths. Further, Levesque et al. [63] found that increased iWUE did not promote tree growth under xeric regions in Italy and mesic areas in Switzerland. Sleen et al. [5] also suggested that increased iWUE did not stimulate tree growth in tropical forests at Bolivia, Cameroon and Thailand. In arid northwest China, some studies also demonstrated that increased iWUE did not always enhance the tree growth [52, 64, 65].

The insignificant contributions of changed iWUE on tree growth may be due to the limited role of water use in tree growth and/or the strong influence of other factors, such as climate, that dominates tree growth. For example, current long term tree-ring width series have still been most widely used to reconstruct past climate change, suggestive of that climate other than iWUE is the major limiting factor for tree growth. It is expected that the influence of iWUE on tree growth can be enhanced when it is coupled with the influences of other climate factors, but weakened when it contradicts with the influences of other factors. In this humid area, the growth of tree-ring width is mainly limited by drought stress that exists in summer. But the iWUE is sensitive to autumn climate, which has limited contribution to ring-width formation. It is also possible that the changed iWUE has contributed to the increase in density of tree-rings as the autumn climate is often sensitive to latewood formation with high density.

We also observed negative correlations between iWUE and tree growth in, e.g. ~in the 1950s-60s for the first-order difference data, which may be due to their contrary responses to stomatal conductance changes (Fig 8). However, the iWUE has a negative response to relative humidity/precipitation instead of a positive one for tree-ring width (S1 Fig and Fig 4b). A dry condition can cause decrease in stomatal conductance and thus low tree growth due to potentially decrease in photosynthesis, which can cause an increase in iWUE according to their definitions (Eq 3). Together, the dominant influences of stomatal conductance can result in negative correlations between tree growth and iWUE. In the 1960s, the sunshine hour was the highest during the past decades (Fig 2e). This may alleviate the limitation of sunshine hours on photosynthesis rate and allows the stomatal conductance to play a more limiting role on iWUE in that period. As both tree growth and iWUE are positively related to photosynthesis, positive relationships between iWUE and tree growth may be due to the control of photosynthesis on both iWUE and tree growth.

## Conclusions

This study presented the first tree-ring width chronology in NML over the period of 1836–2013 and first tree-ring δ^13^C series from 1865 to 2013 in the humid subtropical area in Fujian province, southeast China. Growth of the tree-ring width was mainly limited by summer drought due to the peak temperature and relatively low precipitation. The tree-ring carbon isotope discrimination responded negatively to sunshine hours and positively to relative humidity/precipitation in autumn. The iWUE showed an active response to *c*_a_ before 1970s and a negative response after 2003, resulting in a steady increase/stable in iWUE. The iWUE trends were mainly driven by *c*_a_, and climate factors may be play crucial role in modulating the interannual and decadal variations of iWUE. Our study found the instable relationships between iWUE and tree growth, and concluded that the contribution of iWUE to the tree growth is not significant in most periods. This may be due to the influences of other factors. Our study provided preliminary results on the long term relationships among climate, iWUE and tree growth over the past 150 years for a region in humid subtropical China.

## Supporting information

S1 Fig Correlations between climate variables and (a) intrinsic water-use efficiency (iWUE) and (b) the first-order differenced iWUE from the start of previous growing season (previous February) to the end of current growing season (current December) for the period of 1955–2013. SO represents the combination of data from September to October. The black (green) horizontal dash lines represent the 95% (99%) confidence level.
(TIF)

S1 Table Tree-ring data. (XLSX)
