# Supplementary figures and images for: Climate, intrinsic water-use efficiency and tree growth over the past 150 years in humid subtropical China
Source: PLoS One. 2017 Feb 9;12(2):e0172045. doi: 10.1371/journal.pone.0172045 (PMC5300276; doi:10.1371/journal.pone.0172045)

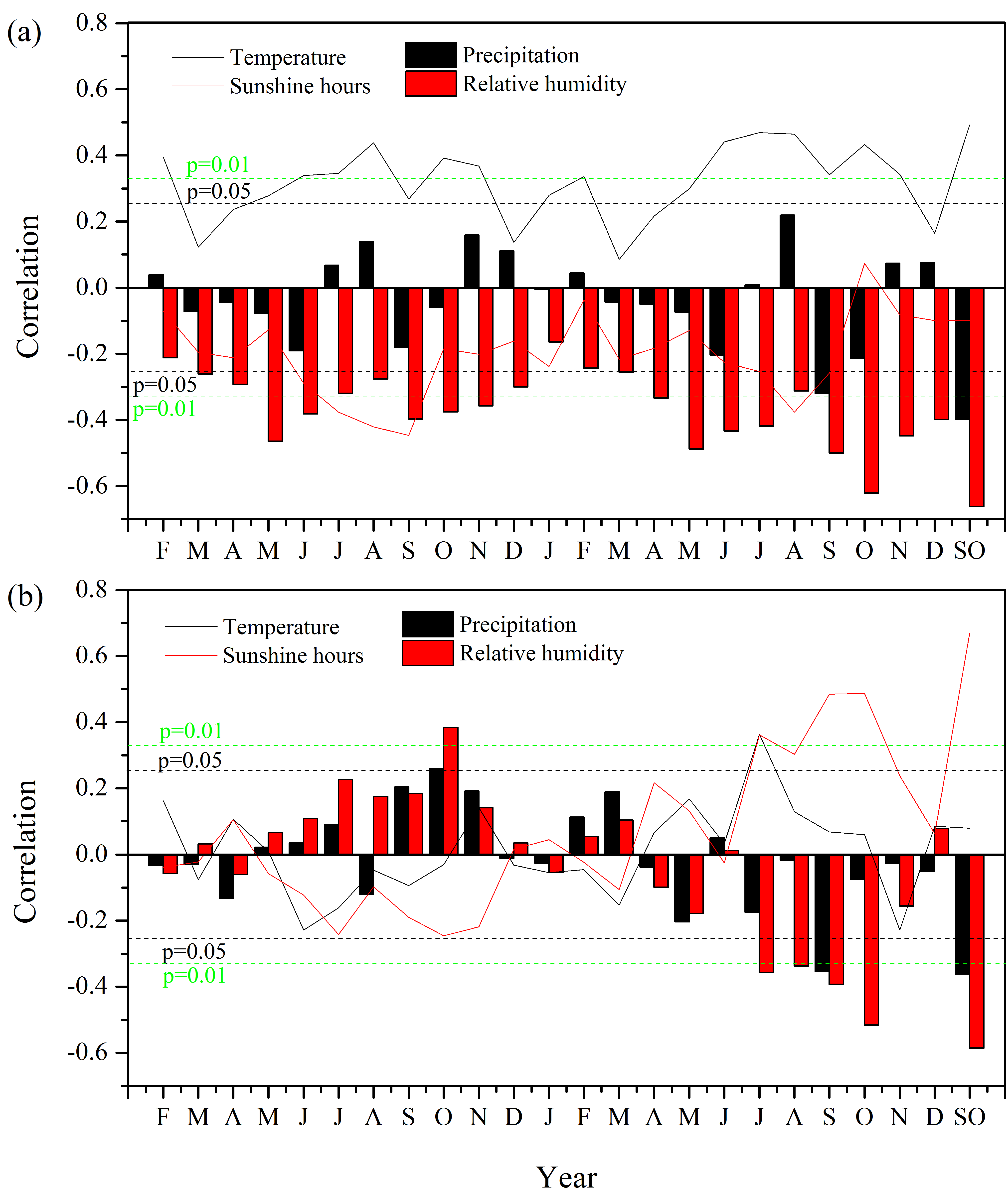

Supplement: S1 Fig — SO represents the combination of data from September to October. The black (green) horizontal dash lines represent the 95% (99%) confidence level. (TIF) [file pone.0172045.s001.tif]
